# 计划性亚肺叶切除治疗T1aN0M0期非小细胞肺癌的进展

**DOI:** 10.3779/j.issn.1009-3419.2010.12.14

**Published:** 2010-12-20

**Authors:** 宝东 刘

**Affiliations:** 100053 北京，首都医科大学肺癌诊疗中心，首都医科大学宣武医院胸外科 Department of Toracic Surgery, Xuanwu Hospital, Afliate to Capital Medical University, Beijing 100053, China

临床T1aN0M0（cIa）期肺癌是指肿瘤最大径≤2 cm，且周围为肺组织或脏层胸膜包绕，气管镜下未见病变侵及叶支气管。按照美国国家癌症综合网络（National Comprehensive Cancer Network, NCCN）^[1]^和美国胸科医师协会（American College of Chest Physicians, ACCP）^[2]^的肺癌治疗指南：Ⅰ期肺癌适合手术者应该进行解剖学肺叶切除加纵隔淋巴结清扫或采样，不适合肺叶切除者应该采用亚肺叶切除（首选肺段切除，次选楔形切除），不适合手术者应该选择射频消融或立体定向放射治疗。

## 1 计划性亚肺叶切除

早期的亚肺叶切除主要用于肺功能低的患者，多数研究^[3-6]^结果提示亚肺叶切除与肺叶切除术相比具有相同的生存率，而死亡风险更低。而对于肺功能正常的早期肺癌患者宜行亚肺叶切除即计划性亚肺叶切除术（intentional sub-lobar resection）。Read等^[7]^于1990年进行了首次尝试：比较了肺叶切除（131例）、肺段切除（107例）和楔形切除（6例）治疗T1N0M0非小细胞肺癌（non-small cell lung cancer, NSCLC），结果显示亚肺叶切除与肺叶切除术的5年生存率无差异。Ginsberg和Rubinstein^[8]^进行随机对照临床试验治疗247例T1N0的早期NSCLC，其中亚肺叶切除122例（包括肺段切除80例和楔形切除42例），肺叶切除125例。亚肺叶肺切除与肺叶切除比较，不能降低围手术期并发症、死亡率和改善术后肺功能，两组5年生存率无统计学差异（*P*=0.062），但是肿瘤特异性死亡率增加47%；局部复发率每人每年肺叶切除为0.019、肺段切除为0.040，楔形切除为0.084，亚肺叶切除是肺叶切除的3倍多（*P*=0.02），亚肺叶切除的肿瘤特异性死亡率增加50%。该研究结果宣布了肺叶切除仍然是T1N0期NSCLC的标准术式，亚肺叶切除是早期低肺功能NSCLC的选择治疗方式。但是，Ginsberg的研究存在争议，如样本量小、长期随访总生存没有统计学差异、 > 2 cm的肿瘤多见、1/3的患者楔形切除。

近年来，随着CT筛查的进步，越来越多的早期小型肺癌（≤2 cm）被发现；同时CT和病理评价肺腺癌，特别是细支气管肺泡癌（bronchioloalveolar carcinoma, BAC）也有一定进展，使计划性亚肺叶切除的地位重新得到肯定。

Kodama等^[9]^回顾性分析了亚肺叶切除对T1N0M0期NSCLC的疗效，肺段切除（46例）与肺叶切除（77例）比较，5年生存率分别为93%和88%，局部复发率分别为8.7%和1.3%。Okada等^[10]^比较了T1N0M0 NSCLC患者接受扩大的肺段切除术（70例）与肺叶切除术（139例）的疗效，5年生存率分别为87.3%和77.7%（*P*=0.164 4）。Koike等^[11]^回顾性评价了74例亚肺叶切除（肺段切除60例和楔形切除14例）和159例肺叶切除治疗Ⅰa期NSCLC的效果，5年生存率分别为89.1%和90.1%，5年无病生存率（disease free survival, DFS）分别为88.7%和89.8%，两组无差异。Kraev等^[12]^回顾性研究了肺叶切除（215例）和楔形切除（74例）治疗Ⅰ期肺癌的多中心研究结果，两组间总生存时间无差异，但是对 < 3 cm的肿瘤，肺叶切除的生存时间明显优于楔形切除（*P*=0.029）。Yamashita等^[13]^回顾了全电视胸腔镜下的肺段切除（38例）和肺叶切除（71例）在Ⅰ期NSCLC的临床结果，提示两组在局部复发率、远处转移率、长期生存方面没有统计学差异。

Okada等^[14]^在亚肺叶切除和肺叶切除治疗T1N0M0的前瞻性多中心非随机研究中，5年DFS分别为85.9%和83.4%，5年生存率分别为89.6%和89.1%。多因素分析证实，亚肺叶切除组的复发和预后不亚于肺叶切除组，而在保护肺功能方面优于肺叶切除组。一项关于比较亚肺叶切除和肺叶切除治疗Ⅰ期NSCLC长期生存的*meta*分析^[15]^显示：总计2 790例患者，两组间1年、3年、5年生存率无统计学差异，分别为0.7%（*P*=0.365 9）、1.9%（*P*=0.508 8）和3.6%（*P*=0.360 3）。

目前正在进行评估肺段切除在≤2 cm早期周围型肺癌中的前瞻性多中心随机对照Ⅲ期临床研究[CALGB]140503。JCOG0802/WJOG4607L^[16]^是一项日本临床肿瘤组和西日本肿瘤组第一项合作课题，比较肺段切除与肺叶切除治疗在≤2 cm早期周围型肺癌总生存非劣效性的Ⅲ期临床试验，开始于2009年8月，3年内71个单位入组共1 100例患者，首要终点为总生存率。

## 2 影响亚肺叶切除疗效的因素

### 2.1 肿瘤大小

Okada等^[17]^复习了1 272例完全切除的NSCLC的临床结果：肿瘤5年特异性生存率在≤1 cm、1.1 cm-2.0 cm、2.1 cm-3.0 cm和 > 3.0 cm四组中分别为100%、83.5%、76.5%和57.9%；病理Ⅰ期NSCLC分别为100%、92.6%、84.1%和76.4%。肺叶切除、肺段切除和楔形切除治疗Ⅰ期NSCLC在肿瘤≤2.0 cm和2.1 cm-3.0 cm两组的5年肿瘤特异性生存率分别为92.4%和87.4%、96.7%和84.6%、85.7%和39.4%。因此肿瘤大小是影响预后和外科治疗计划的独立因子。

Yamato等^[18]^对36例T1N0M0期局限性BAC（Noguchi A/B型）亚肺叶切除随访30个月没有发现肿瘤复发；≤2 cm的腺癌10%存在段淋巴结转移， > 2 cm的腺癌15%存在纵隔淋巴结转移，因此对≤2 cm的腺癌行肺段切除，术中确认有无段淋巴结转移，对≤ 2 cm的腺癌行肺叶切除。

而Patz等^[19]^应用*Cox*比例风险模型回顾性分析了510例Ⅰa期NSCLC，未发现肿瘤大小与生存有关（*P*=0.597），作者认为CT筛查的肿瘤较小的肺癌的生存率并不高，可能是由于提前偏倚所致，需要前瞻性随机研究。

### 2.2 影像学类型

磨玻璃样改变（ground-glass opacity, GGO）是指在HRCT上表现为肺内≤2 cm云雾状密度增高影，在阴影区可见肺血管及支气管纹理的结节；在病理上表现为各种原因引起的炎症、肺纤维化、非典型腺瘤样增生（atypical adenomatous hyperplasia, AAH）和BAC。AAH是肺腺癌的癌前病变，BAC属于特殊类型肺癌，侵袭性较低，临床转归与普通类型肺腺癌有明显的不同。

Kodama等^[20]^将患者按照GGO成分 > 50%和 < 50%两组，依据GGO来判断是否是BAC的敏感性为76%、特异性为95%，两组3年无病生存率分别为100%和72%。Nakata等^[21]^对146例外科切除的T1N0M0期NSCLC按照GGO含量研究其病理及预后：Ⅰ型（90%-100%）的87%为非浸润性BAC，Ⅱ型（50%-89%）的55.6%为腺癌，以上两型无淋巴结转移；而Ⅲ型（10%-49%）的20%和Ⅳ型（< 10%）的24.4%有淋巴结转移；3年无瘤生存Ⅰ/Ⅱ型为97.7%，Ⅲ型为86.1%，Ⅳ型为78.5%。Matsuguma等^[22]^将96例 < 3 cm的NSCLC按照实性成分的比例分为5型：实性成分0为Ⅰ型、1%-25%为Ⅱ型、26%-50%为Ⅲ型、51%-75%为Ⅳ型、76%-100%为Ⅴ型，发现非实性成分 > 50%者无淋巴结转移或复发。

一项关于CT与病理学在评价cⅠa期周围型非侵袭性肺癌符合率的前瞻性多中心临床研究（JCOG0201）发现：肿物在纵隔窗直径与肺窗直径之比≤25%时，CT诊断非侵袭性的特异性为98.7%（95%CI: 93.2%-100%），敏感性为16.2%（95%CI: 11.5%-21.9%）。因此目前日本正在以GGO > 75%为界限，进行大楔形切除治疗早期腺癌的前瞻性多中心Ⅱ期临床单臂研究（JCO G0804/ W JOG4507L）；而JCOG0802/W JOG4607L^[16]^是针对GGO < 75%的病灶进行的比较肺段切除与肺叶切除Ⅲ期临床试验。

### 2.3 病理类型

Noguchi等^[23]^研究了236例手术切除的≤2 cm周围性腺癌的标本，按照肿瘤生长类型分成6种，A型：局部性BAC；B型：局部性BAC伴局部肺泡萎陷，出现肺纤维化；C型：局部性BAC伴有局部纤维母细胞增生；D型：低分化腺癌；E型：管状腺癌；F型：乳头状腺癌。其中A型、B型肺泡上皮被癌细胞代替，无纤维母细胞增生，无淋巴结转移，5年生存率为100%。

Kondo等^[24]^的研究结果提示，Noguchi A型/B型可以进行VATS楔形切除，其它类型需要进行标准肺叶切除。

### 2.4 癌胚抗原（carcinoembr yonic antigen, CEA）水平

Takamochi等^[25]^对189例≤3 cm的周围性肺腺癌分为两组：Ⅰ组50例（肿瘤阴影消失率≥0.8，CEA正常）和Ⅱ组139例（肿瘤阴影消失率 < 0.8，CEA升高），结果两组5年生存率分别为95%和87%，单因素和多因素分析肿瘤阴影消失率和CEA是独立的预后因子。Inoue等^[26]^回顾性分析了143例完全切除的患者，术前CEA水平是一个独立的预后不良的因素，淋巴结转移在胸膜受累或CEA升高者增加， > 1.5 cm者淋巴结转移率为16.9%； > 1.5 cm、胸膜受累、CEA升高建议肺叶切除。

### 2.5 淋巴结转移

Fukui等^[27]^研究认为，肿瘤的大小、分化程度、脏层胸膜的外侵、术前CEA水平、GGO%与淋巴结转移明显相关。既往文献报道：≤2 cm的肿瘤大约20%存在淋巴结转移，其中60%左右为N2转移（表 1）；≤1 cm的肿瘤大约10%存在淋巴结转移（表 2）。Asamura等^[28]^的研究认为，≤2 cm的鳞癌很少出现淋巴结转移。因此Watanabe等^[38]^得出结论：≤2 cm的周围型腺癌需要纵隔和肺门淋巴结的清扫，≤2 cm的周围型鳞癌、≤2 cm的局限性BAC（Noguchi A型/B型）和≤1 cm的周围型腺癌不需要清扫淋巴结。

**1:** 肿瘤≤2 cm的Ⅰa期非小细胞肺癌淋巴结转移情况 Tumor sized 2 cm or smaller in stage Ia nodal disease

Study	*n*	Positive nodes (%)	N2 disease (%)
Asamura (1996)^[28]^	174	20	60
Konaka (1998)^[29]^	171	17.5	66
Takizawa (1998)^[30]^	157	17	-
Wu (2001)^[31]^	103	20.4	-
Watanabe (2002)^[32]^	267	10.8	65.5

**2:** 肿瘤≤1 cm的Ⅰa期非小细胞肺癌淋巴结转移情况 Tumor sized 1 cm or smaller in stage Ⅰa nodal disease

Study	*n*	Positive nodes (%)
Oda (1998)^[33]^	22	0
Suzuki (2001)^[34]^	10	20
Miller (2002)^[35]^	100	7
Moriya (2004)^[36]^	27	11.1
Zhou (2009)^[37]^	41	15

## 3 手术方法

### 3.1 手术术式

手术术式除了与病变本身（大小、部位、影像学、病理学）有关外，主要与有无淋巴结转移有关。一般来说肺段切除需要系统纵隔肺门淋巴结清扫或采样，楔形切除不需要淋巴结采样。肺叶由若干肺段组成，每一肺段都有独自的肺动脉、肺静脉和肺段支气管分布，故肺段切除为解剖性切除，临床上常用于左肺上叶舌段、左肺下叶背段或右肺下叶背段切除术，而肺楔形切除则不是解剖性切除。

近年来随着电视胸腔镜的普及，胸腔镜下肺段切除也逐渐开展起来，但是VATS肺段切除较开胸肺段切除困难得多^[39, 40]^。Tsubota等^[41]^在肺段血管、支气管游离后，如果清扫或采样的淋巴结阳性，则以段间平面的保留侧为界限，应用内镜直线切割缝合器或电刀切除肺段称之为扩大肺段切除术。Okada等^[42]^描述了另一种VATS肺段切除的方法：在纤维支气管镜引导下，喷射通气膨胀切除的肺段，保留的肺段萎陷，在该层面应用电刀切除肺段，肺断面覆盖可吸收纤维蛋白胶。Bando等^[43]^采用肺动脉引导肺段切除的方法治疗T1N0M0肺癌也取得良好效果。

Sienel等^[44]^在一项手术治疗Ⅰa期NSCLC低肺功能患者的回顾性研究中指出：楔形切除的局部复发率明显高于肺段切除（55% *vs* 16%, *P*=0.001），肺段切除的肿瘤特异性5年生存率优于楔形切除（71% *vs* 48%, *P*=0.016），因此开胸肺段切除在生存率和局部复发方面均优于楔形切除。Okada等^[14]^关于VATS亚肺叶切除治疗cT1aN0M0期NSCLC的前瞻性多中心临床研究结果显示肺段切除与楔形切除的5年生存率没有统计学差异。而Yamato等^[45]^回顾分析了523例cT1N0M0期周围性肺腺癌治疗结果：肺段切除治疗≤2 cm肺腺癌的效果明显优于楔形切除（*P*=0.001），但是对于≤1 cm的Noguchi A型/B型肿瘤来说，两种手段的治疗效果无统计学差异。

### 3.2 肿瘤切缘

应用直线切割缝合器切肺时，切割刀对正常肺组织有一个挤压牵拉作用，实际切割的距离要缩小，同时癌细胞对周围组织存在一定的浸润，因此切缘可能存在肿瘤细胞的残留。为此有作者直接用电刀在切割平面切割肺组织，肺断面喷涂可吸收纤维蛋白胶；而对肺气肿的病人需要使用定制的切割缝合器，切除肿瘤侧为单排钉，另一侧为三排钉，以保证足够的切缘^[46]^。术中病理检查对确定切缘十分重要，病理检查方法包括术中切缘细胞学印片检查^[47, 48]^。

El-Sherif等^[49]^回顾了81例NSCLC手术后结果，肺楔形切除切缘 < 1 cm的局部复发率高于≥1 cm者（*P*=0.04）。有研究^[50]^指出：切缘/肿瘤直径之比 > 1与 < 1者相比，明显减少局部复发（25.0% *vs* 6.2%, *P*=0.001 4）。为减少局部复发，Fernando等^[51]^在术中的切割缝合线处覆盖125I粒子网，可以减少局部复发。美国外科医师学会肿瘤学组正在进行亚肺叶切除辅助粒子植入与单纯亚肺叶切除的随机对照试验[ACOSOG]Z4032。

## 4 总结

总之，随着胸部低剂量螺旋CT筛查出大量GGO和肿瘤较小的早期肺癌（≤2 cm），同时肺腺癌特别是BAC比例的增加，计划性亚肺叶切除在肺癌手术的临床地位将越来越受到重视，还需要进行广泛的前瞻性多中心随机对照临床研究。

目前而言，考虑到将来出现肺的复发癌，我们对计划型亚肺叶切除提出以下原则：肺楔形切除的适应症：①肿瘤≤2 cm，临床检查无淋巴结转移；②肿瘤位于周边，利于楔形切除，肿瘤切缘应该在2 cm以上；③GGO% > 75%；④术中病理检查无肿瘤外侵；肺段切除的适应症①肿瘤≤2 cm，临床检查无淋巴结转移；②肿瘤位于肺实质内，保证肿瘤切缘在2 cm；③GGO% > 50%；④术中病理检查无肿瘤外侵；⑤常规肺门和纵隔淋巴结采样或清扫。
